# Canine parvovirus and pseudorabies virus coinfection as a cause of death in a wolf (*Canis lupus*) from southern Italy

**DOI:** 10.1002/vms3.270

**Published:** 2020-04-16

**Authors:** Maria Grazia Amoroso, Denise Di Concilio, Nicola D’Alessio, Vincenzo Veneziano, Giorgio Galiero, Giovanna Fusco

**Affiliations:** ^1^ Department of Animal Health Experimental Zooprophylactic Institute of Southern Italy Portici Italy; ^2^ Department of Veterinary Medicine and Animal Production University of Naples Federico II Naples Italy

**Keywords:** canine parvovirus, *Canis lupus*, pseudorabies virus, sequencing

## Abstract

Pseudorabies virus (PRV) or suid herpesvirus 1 (SHV‐1) is the causative agent of Aujeszky's disease, a highly contagious viral infection which causes neurological fatal illness in mammals other than suids. Here we report a case of a young wolf (*Canis lupus*) of around 2 years found dead by a hunter in the province of Avellino, Campania Region. Necropsy showed pathological findings consistent with encephalitis and gastroenteritis. Organs were analysed by microbiological and molecular investigations following standard procedures to ascertain the possible cause of death. Real‐time PCR revealed the presence of PRV in the brain and of canine parvovirus 2b in organs like intestine, liver, brain, kidney and pancreas. Death probably occurred very shortly after SHV‐1 infection in an animal already weakened by parvovirosis.

## Graphical abstract

We describe a case of wolf dead since infected by pseudorabies virus and canine parvovirus, with pseudorabies attributable to wild boar meat ingestion. Since this virus has been recently described as responsible for human encephalitis, its presence in wild animals must be taken in consideration for the circulation of the virus in the environment.

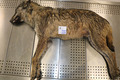

## 1 INTRODUCTION

Pseudorabies (Aujeszky's disease, AD) is a highly contagious disease caused by suid herpesvirus 1 and has significant impact on the economy. The virus is a member of the Herpesviridae family, subfamily Alphaherpesvirinae (Fonseca et al., 2010). AD infects pigs worldwide, and the clinical signs of the disease varying with the age of the animal, include respiratory and reproductive symptoms (Pomeranz, Reynolds, & Hengartner, 2005; Sozzi et al., 2014). Moreover, like the other herpesviruses, SHV‐1 is neurotropic and can infect the nervous system by trans‐synaptic passage and retrograde axonal transportation (Pomeranz et al., 2005). Animals belonging to the Suidae family are the natural hosts of PRV: domestic pigs and wild boars are the only animals which can survive the infection becoming reservoir of the virus (Sozzi et al., 2014). The disease is also described in other domestic (goats, sheep, dogs, cats, horses and bovines) as well as in wild (bears, coyotes and foxes) mammals, which are all lethally infected by direct contact with suids or by ingesting their meat (Moreno et al., 2015; Verpoest, Cay, Bertrand, Salumont, & Regge, 2014). Recent reports (Ai et al., 2018; Yang et al., 2019; Zhao et al., 2018) have demonstrated that SHV‐1 can cause encephalitis and endophthalmitis also in humans, which had been thought to be refractory to the infection. All the reported patients had jobs involving the handling of pigs, reinforcing the importance of animal vaccination against the virus. Even though extensive vaccination programmes have led to the eradication of the disease in domestic pigs in many European countries, the virus still circulates in wild boars population (Lari et al., 2006; Verin, Varuzza, Mazzei, & Poli, 2014; Verpoest et al., 2014) as witnessed by surveillance studies as well as by not infrequent contamination of dogs used for wild boar hunting (Moreno et al., 2015; Steinrigl et al., 2012). The hunters have indeed the wrong habit to repay their dogs by giving them entrails of the wild boars hunted. Canids show a very serious nervous symptomatology such as tremors, laringe and faringe muscle spasms, vomiting, very often accompanied by intense pruritus with death occurring within 24–48 hr after appearance of first symptoms (Moreno et al., 2015). Furthermore, wild boars represent a risk not only for hunting dogs, but also for wildlife species (foxes, wolves, bears) living in the same areas, especially if habitually consuming their meat. In this paper, we describe the presence of SHV‐1 in the brain of a wolf found dead in the province of Avellino, Campania region, Southern Italy. The virus has been identified by real‐time PCR and confirmed by sequencing analysis. So far, only another case of AD in wolf described in Europe is in Belgium in 2014.

## 2 MATERIALS AND METHODS

### 2.1 Materials

In December 2018, a young wolf (*Canis lupus*) of around 2 years was found dead by a hunter in the province of Avellino, Campania Region and referred for necropsy to our laboratories. SHV‐1 positive control, strain ‘Kaplan’ was kindly given by Friederich‐Loeffler Institut, Federal Research Institute for Animal Health, Federal Republic of Germany. Parvovirus strains, a, b and c used as positive controls, were kindly given by University of Bari Aldo Moro, Italy.

### 2.2 Bacteriological analysis

Aerobic and anaerobic cultures of brain, liver, lymph nodes, spleen, kidneys and intestine were carried out following standard procedures to determine the possible cause of death.

### 2.3 Viral Nucleic acids extraction procedures

Each organ (25 mg of tissue in 1 ml phosphate‐buffered saline solution) was homogenized by Tissue Lyser (Qiagen). Nucleic acid extraction was carried from 400 µl of organ homogenate by using a Qiasimphony automated extraction system (Qiagen) with the DSP Virus/Pathogen Midi kit (Qiagen) according to the manufacturer's instructions. Nucleic acids were eluted in 60 µl of elution buffer containing 40 unit/µl RNase inhibitor (Promega) and immediately analysed using real‐time PCR. A strain of SHV‐1 isolated in our laboratory from the brain of a farm dog, was also analysed for phylogenetic analysis purposes. The dog, dead with AD in 2012, lived in a pig farm in the province of Sorrento (Campania Region). Viral DNA was extracted from cell culture (RK13 cells) supernatant (400 µl) as indicated above.

### 2.4 Real‐time virus identification

DNA viruses (canine herpesvirus type 1, canine parvovirus, suid herpesvirus 1) were investigated by real‐time PCR using a Quantitect Real time PCR detection kit (Qiagen). RNA viruses like: canine adenovirus 1 and 2, morbillivirus (also known as canine distemper virus) and canine coronavirus were investigated by real‐time reverse transcription PCR using AGPATH reaction kit (Thermo Fisher Scientific). All the reactions were carried with primers (Tema Ricerca) and probes (Thermo Fisher Scientific) specific for the virus tested on a Quantstudio5 system (Thermofisher) with protocols routinely used in our laboratory. In particular, SHV‐1 was identified as described by Ma et al. (2008). Canine parvovirus strain identification was carried out by real‐time multiplex PCR as described by Decaro et al. (2010).

### 2.5 SHV sequencing

In order to characterize the strains of suid herpesvirus, 5 µl of nucleic acids underwent PCR to amplify an 804‐bp fragment of the pseudorabies gC gene (Muller et al., 2010). The reaction was carried with Phusion high‐fidelity PCR master mix (Thermofisher), 0.5 µM of each primer and 3% dimethilsulfoxyde with the following thermal profile: 98°C for 30 s, 35 cycles of 98°C for 10 s, 67°C for 30 s, 72°C for 30 s and a final elongation step of 72°C for 10 min. PCR products were analysed by Tape station (Agilent) using the D 5,000 kit.

Amplicons were sequenced by capillary electrophoresis as previously described (Amoroso et al., 2013) The nucleotide sequence similarity searches were performed using the BLAST server (http://www.ncbi.nlm.nih.gov/genbank/index.html) and phylogenetic analysis was carried out using the Jalview and MEGA 7 softwares.

## 3 RESULTS

### 3.1 Necroscopical findings

**Figure 1:**
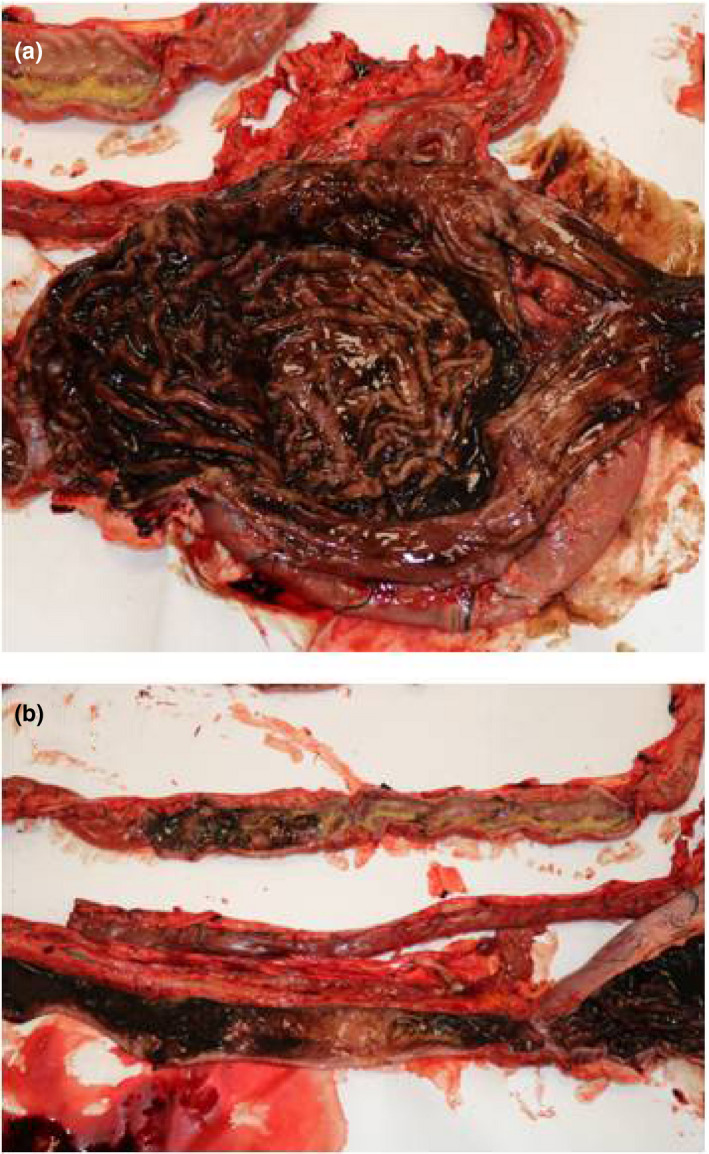
Gross pathology findings. (a) Lumen of the stomach with congested mucosa and the presence of black liquid. (b) Wrinkled intestinal loops with marked vascularization with foci of mucosal consistence mixed with blackish‐coloured material

**Figure 2:**
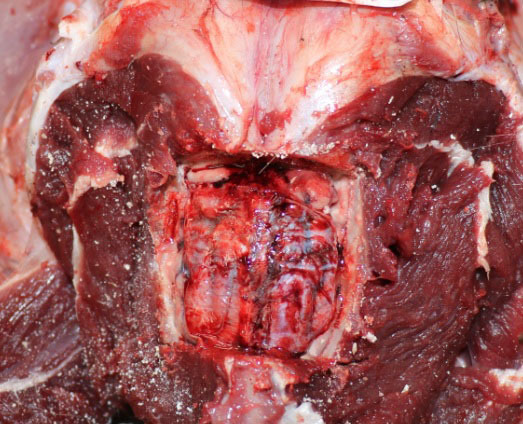
Section of the brain. Modest thickening of the meningeal tissue and marked vascularization are visible

Necroscopy of the wolf revealed gastric wall hyperaemia, the presence of blackish dense fluid in the stomach and the absence of food material (Figure 1a). Oesophagus was markedly congested. In the abdominal cavity, there was congestion of various organs. Pancreas was found haemorrhagic. Analysis of kidneys revealed marked congestion of the renal parenchyma with loss of corticomedullary delimitation. The intestine was characterized by wrinkled loops and marked vascularization (Figure 1b). In the intestine, there was scarce faecal material of mucous consistency mixed with black material, condition indicative of a possible enteritis. Analysis of brain showed modest thickening of the meningeal tissue and evident vascularization (Figure 2), a clinical framework consistent with meningoencephalitis.

### 3.2 Microbiology and virology

Microbiological analysis revealed the absence of pathogenic bacteria in all the organs. Virological investigations gave interesting results. All the organs resulted negative for the following viruses: canine herpesvirus type 1, canine adenovirus 1 and 2, canine distemper virus, canine coronavirus. Brain was positive for the presence of SHV‐1, while intestine and other organs (heart, liver, spleen and brain) were positive for the presence of canine parvovirus (CPV), identified by multiplex PCR further investigation as CPV strain 2b. Any attempt to cell isolate the pseudorabies virus from the samples was unsuccessful probably due to not enough quantity of virus or to the bad condition of the material used.

### 3.3 Sequencing analysis of SHV‐1

**Figure 3:**
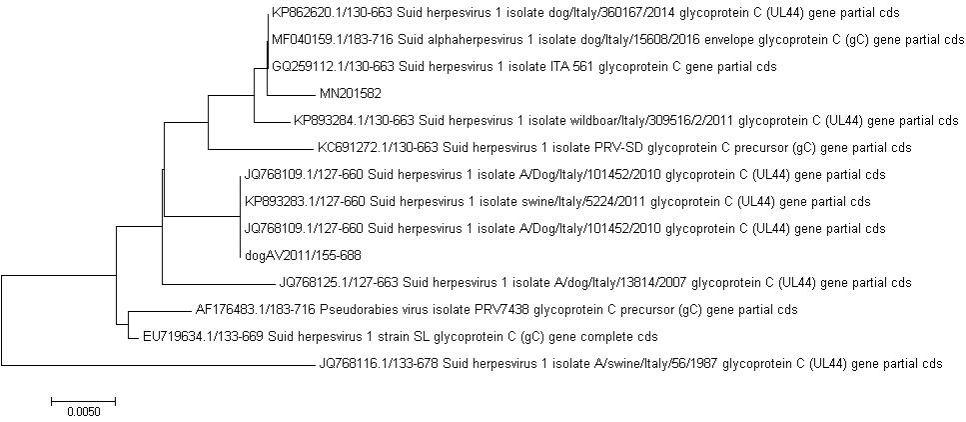
Phylogenetic analysis based on the partial nucleotide sequence (804 bp fragment) of the pseudorabies gC gene (UL44). The evolutionary history was inferred using the neighbour‐joining method (Saitou & Nei, 1987). The optimal tree with the sum of branch length = 0.08126798 is shown. The tree is drawn to scale, with branch lengths in the same units as those of the evolutionary distances used to infer the phylogenetic tree. Evolutionary analyses were conducted in MEGA7 (Kumar, Stecher, & Tamura, 2016). MN201582 (marked with an asterisk) indicates the sequence obtained in the present study

Partial UL44 gene sequencing was carried out on both the SHV‐1 strains investigated in the present study. Sequence of the strain from the Sorrento farm dog (2012) exhibited 100% homology with a strain identified in a *Sus scrofa* in 2011 (Gene Bank Accession Number KP893283, Moreno et al., 2015). Phylogenetic analysis of the SHV‐1 identified in wolf (AN: MN201582) showed the highest homology (only 2 nucleotide of difference) with a strain (AN: KP862620.1, Moreno et al., 2015) isolated in 2014 in a hunting dog from Forli (Emilia Romagna Region, Italy) and with a strain (AN: GQ259112) identified in 1993 in a wild boar from Abruzzi Region (Muller et al., 2010) (Figure 3). SHV‐1 strains from the wolf and from the farm dog, exhibited 8 nucleotide differences (98.5% identity).

## 4 DISCUSSION

**Figure 4:**
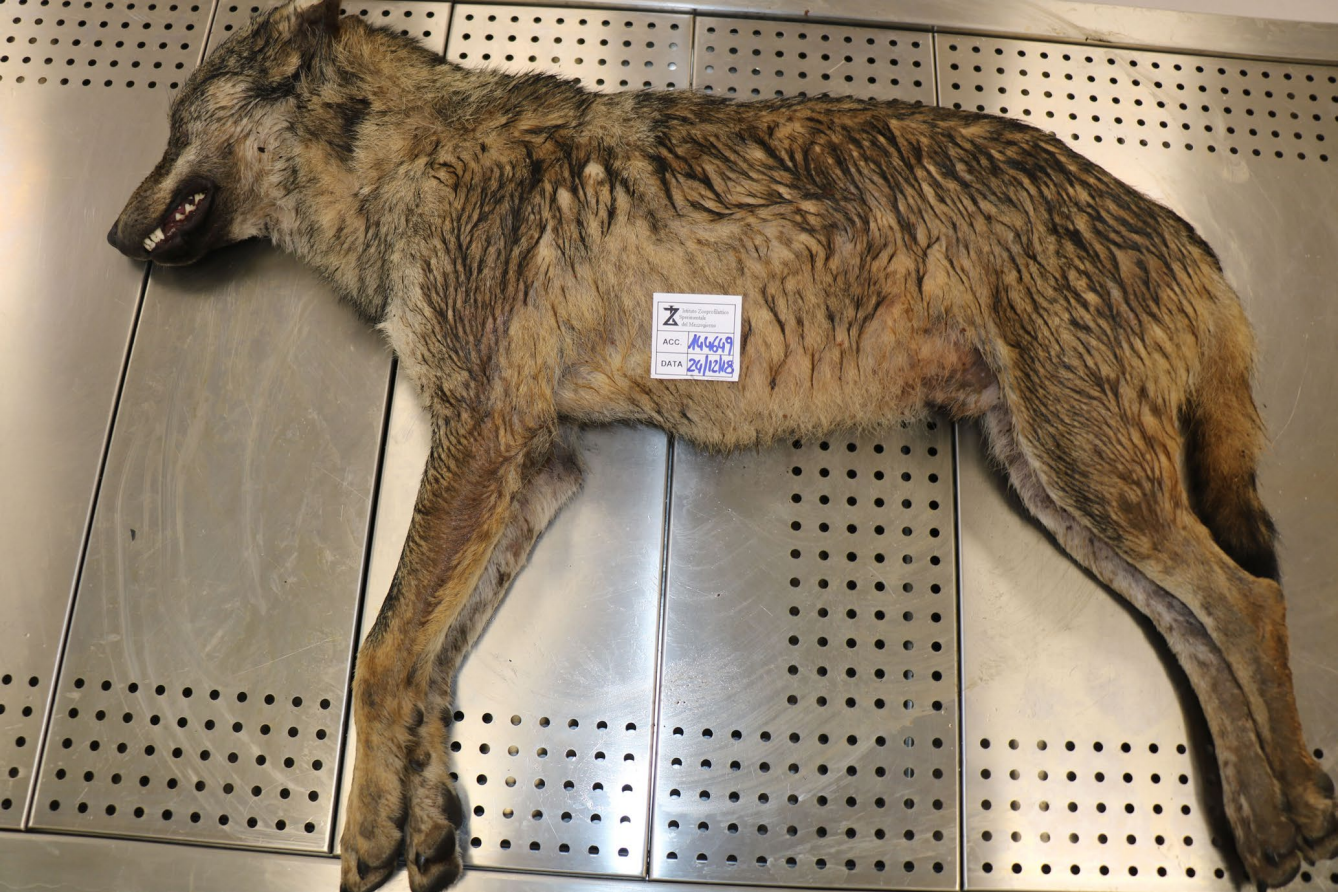
*Canis lupus* necroscopy. Skeletal and muscular development in the norm. Good nutritional status. Uniform coat with abundant winter undercoat; mantle with predominantly grey‐tawny tones and the partial presence of black bands on the front legs. The absence of signs of rubbing typical for AD in canids

Pseudorabies virus has been detected in many domestic and wild mammals (Moreno et al., 2015; Verpoest et al., 2014) as well as in humans (Ai et al., 2018; Yang et al., 2019; Zhao et al., 2018), witnessing its wide distribution and the ability of the virus to infect numerous species. In this study, we described the case of a wolf simultaneously infected by SHV‐1 and CPV. As to the causes of dead, anatomopathological findings would suggest a multiorgan infection by parvovirus, while the presence of SHV‐1 in the brain would be consistent with AD. In this regard, since the animal was found dead, it had been not possible to establish if it was affected by nervous symptomatology typical of AD in canids. As a matter of fact, the animal did not show any characteristic sign of rubbing (Figure 4). It was therefore possible to hypothesize that the contemporaneous presence of the two viruses (SHV‐1 and CPV) could have led to an more rapid death of the wolf: the course of the pseudorabies could have been accelerated by a general weakened state caused by parvovirosis. The animal could have therefore dead before the onset of the classical symptomatology. In any case, it has also been reported that not all the canids show the same symptoms (www.cfsph.iastate.edu). Results of the sequencing analysis surprisingly showed that the strain identified in the present study (Campania region, 2018) was more similar to strains identified in different years in other regions (Abruzzi 1993 and Emilia Romagna 2014) with respect to the strain isolated from the Sorrento farm dog in 2012 and analysed in the present study for phylogenetic purposes since belonging to the same region. Our results clearly indicate that the difference observed cannot be ascribed to the year of isolation (considering the similarity between strain of 2018 and strain of 1993), nor to the geographical distance, since even if the two cases here reported come from different areas of the same Region (respectively Sorrento and Avellino, around 100 km distant); these places are, however, closer with respect to the other regions to which strains KP862620.1 and GQ259112 belonged.

The difference observed can be most likely explained with the diverse source of contamination: while farm dog was surely contaminated by pigs, wolf was probably infected ingesting wild boar meat. As a matter of fact, wild boar population in Italy is very abundant and wolves consume their meat as a usual part of their diet (Verpoest et al., 2014). Wolf SHV‐1 strain looked indeed more similar to the strains from Abruzzi and Emilia Romagna, (both traced back to wild boars) with respect to the strain from the farm dog, traced back to pigs. Our results seem to confirm the hypothesis of Moreno et al. (2015) according to which there is a divergence between the SHV‐1 strains from wild boars or from animals contaminated by their meats (like the wolf of the present study) and the strains circulating in domestic pigs and in animals in contact with them (like the farm dog of the present study). Our finding represents the second report of PRV in wolf (the first in Belgium in 2014, Verpoest et al., 2014) and confirms the susceptibility of the species to this virus, suggesting possible implications between the presence of wild boar on our territories and conservation of species like wolf in areas where its population is particularly at risk of extinction (Verpoest et al., 2014) or strong reduction with consequent negative impacts on biodiversity. Last but not least, considering the recent reports on human susceptibility to SHV‐1 (Yang et al., 2019), AD could be considered a full‐fledged zoonosis, and therefore much attention should be paid to the control of wild boar population. These animals’ play a potential role in maintaining the virus in the environment and in pig farms, which must be seen also as a possible threat to humans, especially those working in contact with pigs.

## CONFLICT OF INTEREST

The authors declare that they have no conflicts of interest.

## AUTHOR CONTRIBUTION

**Maria Grazia Amoroso:** Conceptualization; Data curation; Formal analysis; Investigation; Methodology; Supervision; Writing‐original draft; Writing‐review & editing. **Denise Di Concilio:** Formal analysis; Investigation; Validation. **Nicola D'Alessio:** Conceptualization; Formal analysis; Funding acquisition; Writing‐original draft. **Vincenzo Veneziano:** Conceptualization; Funding acquisition; Supervision; Visualization. **Giorgio Galiero:** Conceptualization; Data curation; Supervision; Writing‐review & editing. **Giovanna Fusco:** Project administration; Resources; Supervision; Validation.
